# Targeted Gene Therapy of Xeroderma Pigmentosum Cells Using Meganuclease and TALEN™

**DOI:** 10.1371/journal.pone.0078678

**Published:** 2013-11-13

**Authors:** Aurélie Dupuy, Julien Valton, Sophie Leduc, Jacques Armier, Roman Galetto, Agnès Gouble, Céline Lebuhotel, Anne Stary, Frédéric Pâques, Philippe Duchateau, Alain Sarasin, Fayza Daboussi

**Affiliations:** 1 Cellectis S.A., Paris, France; 2 Unité mixte de recherche 8200, Institut Gustave Roussy, Villejuif, France; 3 Cellectis Therapeutics, Paris, France; New England Biolabs, Inc., United States of America

## Abstract

Xeroderma pigmentosum group C (XP-C) is a rare human syndrome characterized by hypersensitivity to UV light and a dramatic predisposition to skin neoplasms. XP-C cells are deficient in the nucleotide excision repair (NER) pathway, a complex process involved in the recognition and removal of DNA lesions. Several XPC mutations have been described, including a founder mutation in North African patients involving the deletion of a TG dinucleotide (ΔTG) located in the middle of exon 9. This deletion leads to the expression of an inactive truncated XPC protein, normally involved in the first step of NER. New approaches used for gene correction are based on the ability of engineered nucleases such as Meganucleases, Zinc-Finger nucleases or TALE nucleases to accurately generate a double strand break at a specific locus and promote correction by homologous recombination through the insertion of an exogenous DNA repair matrix. Here, we describe the targeted correction of the ΔTG mutation in XP-C cells using engineered meganuclease and TALEN™. The methylated status of the *XPC* locus, known to inhibit both of these nuclease activities, led us to adapt our experimental design to optimize their *in vivo* efficacies. We show that demethylating treatment as well as the use of TALEN™ insensitive to CpG methylation enable successful correction of the ΔTG mutation. Such genetic correction leads to re-expression of the full-length XPC protein and to the recovery of NER capacity, attested by UV-C resistance of the corrected cells. Overall, we demonstrate that nuclease-based targeted approaches offer reliable and efficient strategies for gene correction.

## Introduction

Xeroderma pigmentosum (XP) is a rare, autosomal, recessive syndrome characterized by hypersensitivity to UV light [1]. It is also associated with a dramatic predisposition to skin neoplasms. Thus, risk of melanoma and non-melanoma skin cancers has been reported to be increased 2 to 10 thousand-fold, respectively [2]. XP cells are deficient in the nucleotide excision repair (NER) pathway, a complex process involved in the recognition and removal of DNA lesions induced by UV light (cyclobutane pyrimidine dimers and pyrimidine 6-4 pyrimidone photoproducts) [3]. Seven different genes named *XPA* to *XPG* are involved in that process. Mutations within the *XPC* gene are by far the most common genetic alteration found in European and North African XP patients. Among the known genetic alterations, a founder mutation within exon 9 has been described in almost 90% of Maghrebian XP-C patients [4] and corresponds to the deletion of a TG dinucleotide leading to the expression of an inactive and undetectable XPC truncated protein. This lack of NER activity allows UV-dependent DNA damage to accumulate and is responsible for the development of high numbers of skin cancers. Today, there is no curative treatment for XP-C patients and their cancer-free survival relies solely on full body protection from light and/or surgical resections of skin tumors. Autologous grafts have been performed using UV sensitive cells, but the benefit of such treatment is transient [5]. A major advance in cancer prevention would be to engraft patient skin produced *ex vivo* with cells corrected for XPC mutation.

Recently, the stable trans-complementation of *XPC* deficiency has been reported [6]. Using a retrovirus-based strategy, Warrick *et al*. were able to transduce the wild-type *XPC* gene into human primary XP-C keratinocyte stem cells and reconstitute their full NER capacity resulting in UV resistance. Although successfully validated *in vivo* and in a relevant cell line, this complementation strategy is nonetheless liable to generate potential adverse effects due to uncontrolled random integrations of the transgene. Indeed, these undesirable effects have been reported in several complemented cells for disease treatment, especially in the hematopoietic system [7], [8]. In view of this result, genetically modified skin could lead to skin tumor development following engraftment. In addition, because of the ectopic expression of the *XPC* transgene, this strategy prevents physiological regulations of the *XPC* transcription, the importance of which has been described in other studies [9], [10]. Thus, an alternative and safer approach to curing XP-C defective cells is highly desirable.

In the past few years, several studies have demonstrated the tremendous potential of nuclease-based targeted approaches for gene correction [11]–[14]. These approaches rely on the ability of engineered nucleases known as Meganucleases, Zinc Finger nucleases, and TALE nucleases to generate a precise double-strand break at a specific locus and promote targeted homologous recombination (HR) with an exogenous DNA repair matrix [15]–[17].

In this study, we used engineered meganuclease and TALE nuclease to promote the targeted correction of XPC mutation in the XP4PA cell line, which carries the homozygote ΔTG mutation in the *XPC* gene. The presence of methylated cytosines (5 mCs) in the *XPC* locus led us to adapt the design of these tools, as well as our experimental conditions, to optimize their *in vivo* efficacy. We showed that treatment with a demethylating agent or the use of 5 mC insensitive nuclease allowed successuful *XPC* gene correction without requiring selection marker. Such genetic correction enabled re-expression of the full-length XPC protein and full recovery of wild-type UV resistance in the XP4PA cell line. We demonstrate that nuclease-based targeted approaches constitute a robust and reliable strategy for *XPC* gene therapy.

## Results

The recent development of engineered nucleases able to introduce a DSB and stimulate HR at a specific locus [18], [19] has opened up new opportunities for *XPC* gene correction. In order to induce a high frequency of HR at an endogenous locus, it is crucial to generate specific and efficient nucleases. For this study, two types of engineered nucleases were developed to target a DNA sequence located 100 bp upstream from the ΔTG XPC founder mutation (Figure S1A). The first nuclease was a single-chain meganuclease named XPCm, derived from I-CreI endonuclease [15]. The second was a TALEN™ named XPCT1 and derived from TALE AvrBs3 [20].

### An Engineered Meganuclease (XPCm) Specifically Designed to Target the *XPC* Locus

The engineered meganuclease XPCm has been previously described [15]. Its intrinsic activity was first determined by a single-strand annealing (SSA) extrachromosomal assay in CHO-K1 cells (Materials and Methods S1) [21]. XPCm showed high activity similar to that of the meganuclease RAG1m, used here as a positive control, and better than that of I-SceIm (Figure S1B). We then assessed the ability of XPCm to cleave the endogenous XPCt sequence in 293-H cells by quantifying the frequency of targeted mutagenesis (TM) induced by XPCm at its endogenous locus. TM consists of small insertions or deletions of nucleotides resulting from imprecise non-homologous end joining (NHEJ) occurring at the DSB site. TM was quantified by specific PCR surrounding the locus of interest followed by deep sequencing.

We found that XPCm induced weak TM, as the frequency was 3-fold lower than that induced by the RAG1 m meganuclease (Figure S1C). This low efficiency was also observed in the frequency of homologous gene targeting (HGT), with 8 times more events using RAG1 m versus XPCm (Figure S1D). We recently demonstrated the inhibitory effect of DNA target methylation on meganuclease activity *in vivo* [22]. Interestingly, XPCt DNA sequence contained two fully methylated CpG dinucleotides (Figure S2A). We thus hypothesized that methylation of XPCt could prevent XPCm from processing its endogenous locus.

### The Efficiency of XPCm is Strongly Influenced by the Epigenetic Status of its DNA Target

**Figure 1:**
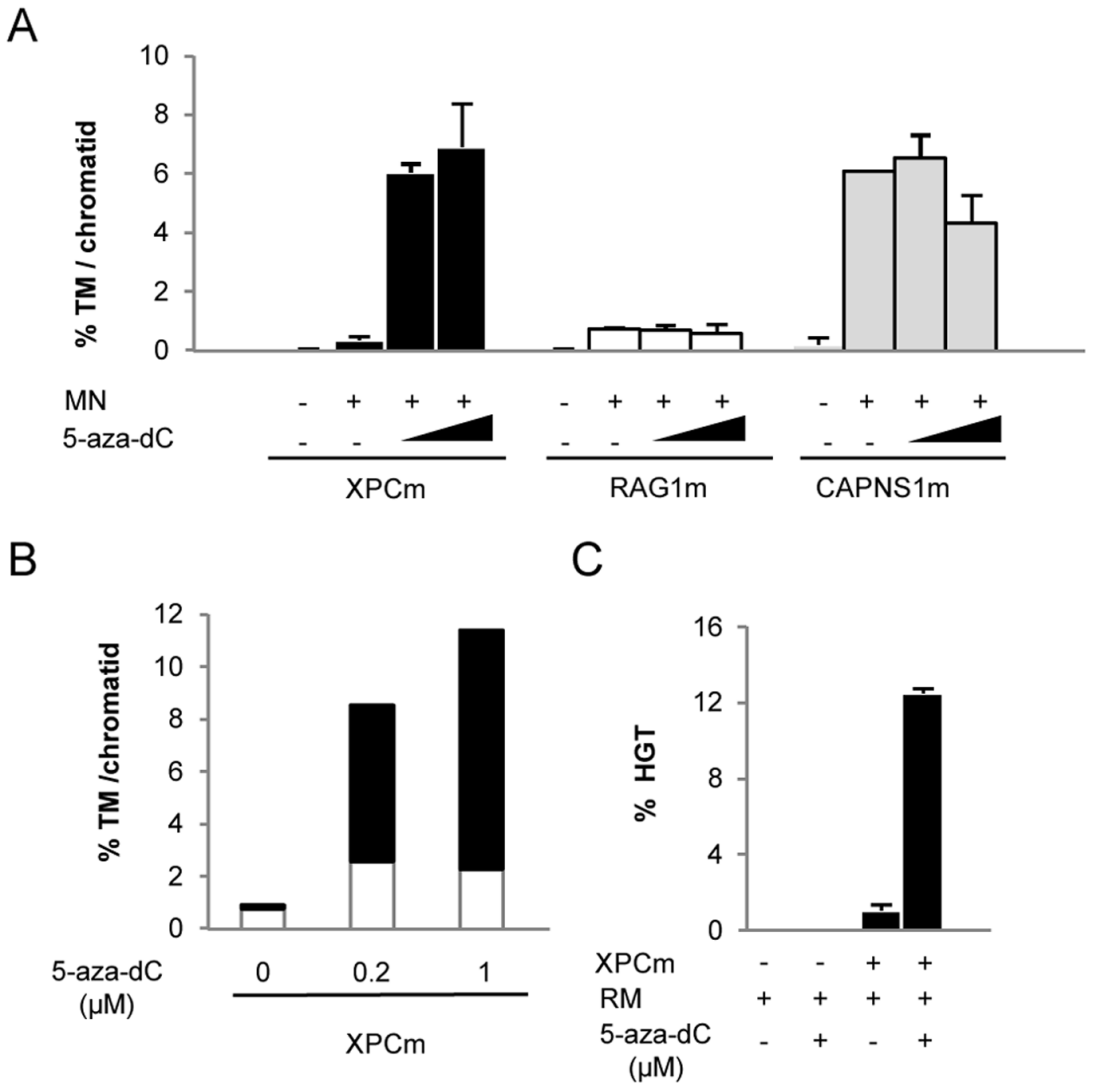
Impact of demethylating treatment on targeted mutagenesis (TM) and homologous gene targeting (HGT) frequencies induced by engineered meganucleases in 293-H cells. (**A**) TM frequency was determined from cells grown with or without 0.2 µM or 1 µM 5-aza-dC and transfected with the engineered meganuclease (MN) XPCm, or RAG1 m and CAPNS1 m, two meganucleases targeting DNA sequences that lack methylated CpG. (**B**) Distribution of TM events in methylated (white) and unmethylated (black) sequences from cells transfected with XPCm with and without 5-aza-dC. (**C**) HGT frequency was determined from cells grown with 0.2 µM (+) or without (−) 5-aza-dC and co-transfected with the DNA repair matrix (RM) and the XPCm engineered meganuclease (+) or empty vector (−).

The demethylating agent 5-aza-dC can be used to overcome the negative impact of CpG methylation on both TM and HGT events induced by nucleases [20], [22]. To check whether it was possible to enhance XPCm-assisted TM and HGT on the *XPC* gene target, 293-H cells were treated with 0.2 or 1 µM 5-aza-dC. Bisulfite treatment of genomic DNA followed by DNA sequencing showed that such treatment induced 35% and 50% demethylation, respectively, of the XPCt target in the cell population (Figure S2A and S2B). Furthermore, TM frequency was substantially increased (16-fold), reaching 6% for both 5-aza-dC concentrations (P<0.001) (Figure 1A).

A similar TM frequency enhancement (25-fold) was observed when the DNA methyltransferase 1 gene was knocked down using specific siRNA (siDNMT1, data not shown). Interestingly, the distribution of TM events within the amplicon population was strongly biased toward demethylated sequences. Up to 80% of mutated events were found within the demethylated DNA sequence population which strongly suggests that demethylation increases the XPCm efficiency at its endogenous locus (P<0.001) (Figure 1B). This conclusion is supported by the fact that no induction of targeted mutagenesis after 5-aza-dC treatment was observed in the cells transfected with meganucleases CAPNS1m and RAG1 m, both targeting unmethylated sequences (P = 0.77) (Figure 1A). In order to determine whether demethylating treatment directly affected the cleavage activity of the nuclease, we used LM-PCR to monitor the non-processed DNA ends generated upon cleavage by the meganuclease (Materials and Methods S1). The number of free DNA ends at the *XPC* locus was increased up to 7-fold in the presence of 5-aza-dC versus untreated cells (P<0.05) (Figure S2C). Although our protocol can only quantify non-processed DNA ends, this result strongly suggests that at least a substantial portion of TM stimulation results directly from higher cleavage activity of the meganuclease on unmethylated sequences.

We then evaluated the impact of 5-aza-dC on the ability of XPCm to trigger HGT in 293-H cells. The DNA repair matrix was composed of two homology arms interrupted by an exogenous DNA sequence (29 bp) specifically designed to screen and identify the HGT events by PCR. Because 5-aza-dC had a major impact on cellular proliferation at a dose of 1 µM, this experiment was performed with dose of 0.2 µM. As regards TM, our results showed that 5-aza-dC treatment increased HGT frequency 12-fold, leading to up to 12% of corrected cells (P<0.001) (Figure 1C). Altogether, our data indicated that the presence of an epigenetic effector such as a 5-aza-dC significantly enhanced meganuclease-assisted TM and HGT at the *XPC* locus.

### *XPC* Gene Correction in XP4PA Cells using XPCm Meganuclease and a Demethylating Agent

**Figure 2:**
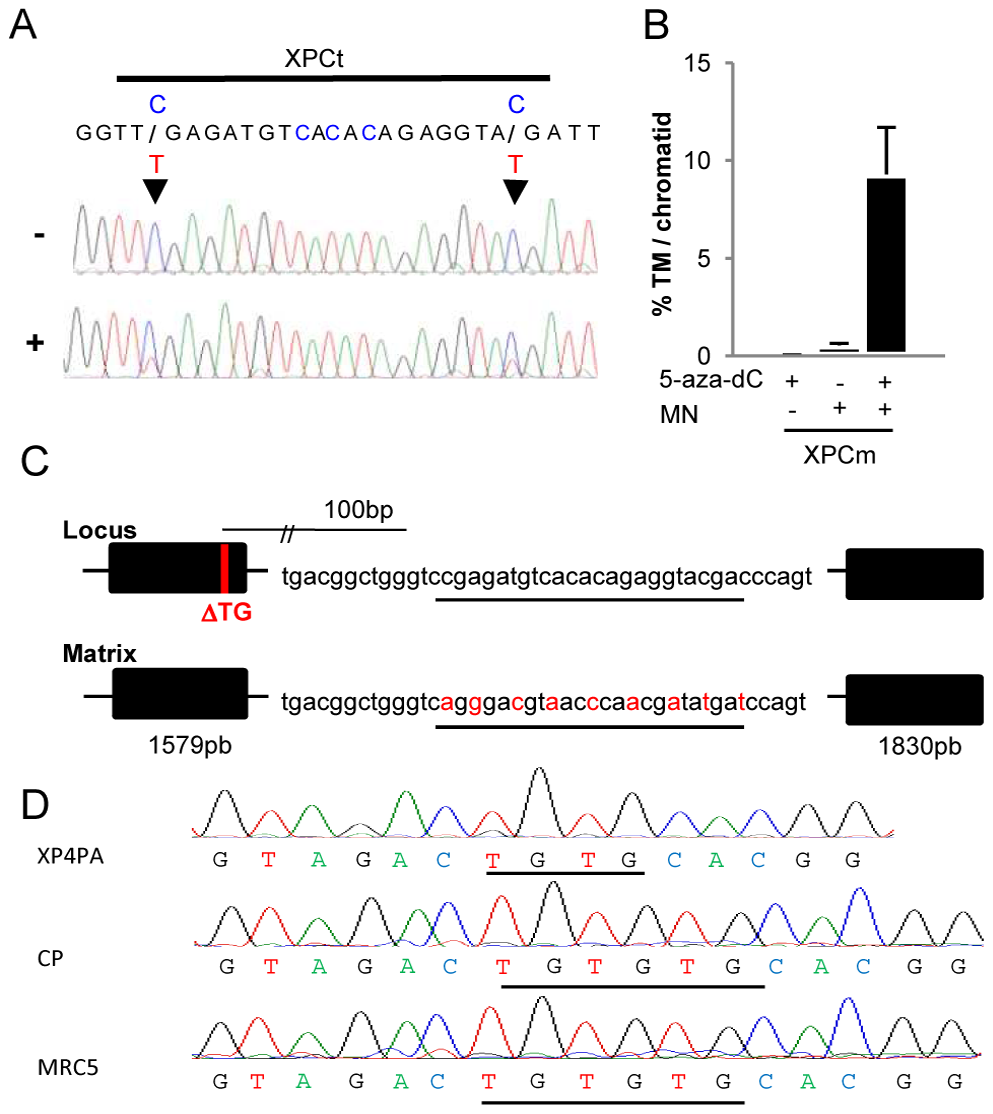
Efficacy of XPCm in XP4PA cells after 5-aza-dC treatment. (**A**) Chromatogram showing the impact of 5-aza-dC treatment on methylating status of the XPCt. Cells were grown with 0.2 µM (+) or without (−) 5-aza-dC and transfected with empty vector under the same conditions as in TM or HGT expriments. While the CpGs present in XPCt was fully methylated under non-treated conditions, the 5-aza-dC treatment induced partial demethylation as shown by the presence of a double peak. (**B**) TM frequency was determined from XP4PA cells grown with 0.2 µM (+) or without (−) 5-aza-dC and transfected with XPCm (+) or empty vector (−). (**C**) Design of the DNA correction matrix used for HGT experiments, which was composed of two arms of 1,579 bp and 1,830 bp, homologous to the *XPC* sequences and separated by the underlined meganuclease-recognizing site (part of the normal wild type sequence of *XPC*). The DNA sequence that was recognized by the meganuclease was modified by producing silent mutations (in red letters) to avoid any cleavage of the matrix by XPCm. (**D**) Sequencing of HGT-PCR products from one corrected population (CP). These sequences were compared to the sequences obtained in the MRC5 cell proficient for XPC (+) and in the parental cell line XP4PA carring the TG deletion (−).

The XP4PA cell line was derived from dermal fibroblast obtained from a patient bearing the homozygote mutation matching the TG deletion in exon 9 of the *XPC* gene [23]. These cells’ impairment in NER has been already described, as well as the possibility of complementation using plasmid or recombinant retroviruses expressing wild type XPC cDNA [24]. As in the 293-H cell line, the XPCt DNA sequence appeared to be fully methylated in XP4PA cells. Treatment of the cells with 0.2 µM 5-aza-dC led to 50% demethylation of XPCt (Figure 2A) and increased TM frequency 18-fold with respect to untreated cells (P<0.05) (Figure 2B). In order to perform gene correction experiments, we designed a new DNA matrix able to restore the *XPC* open reading frame. It contained two arms of 1.5 and 1.8 Kb respectively, homologous to the wild type *XPC* sequences surrounding the cleavage site. To avoid any possible cleavage of the DNA repair matrix by XPCm, silent mutations were introduced within the meganuclease-recognizing site (Figure 2C). XP4PA cells were transfected with the DNA repair matrix and the meganuclease XPCm expression vector. Three days later, cells were seeded at a density of 100 cells per well in a 96-well plate. Each well was then screened for locus-specific integration using specific primers. While no positive wells were found in untreated conditions, 3 out of 480 wells were identified as positive after 5-aza-dC treatment. Further DNA sequencing confirmed that the initial XPC mutation was corrected in 2 out of these 3 cell populations (Figure 2D). Furthermore, the presence of all silent mutations, present only on the matrix plasmid, together with the corrected mutation (100 bp from cleavage site) indicated that these were transferred from the DNA matrix to the genomic DNA, and confirmed the homologous recombination process (data not shown). One corrected population was then sub-cloned. After amplification, we isolated three clones positive for the PCR-detected HGT event and used them for further phenotypic characterizations.

### *XPC* Gene Correction in XP4PA Cells using XPCT1 TALEN™

**Figure 3:**
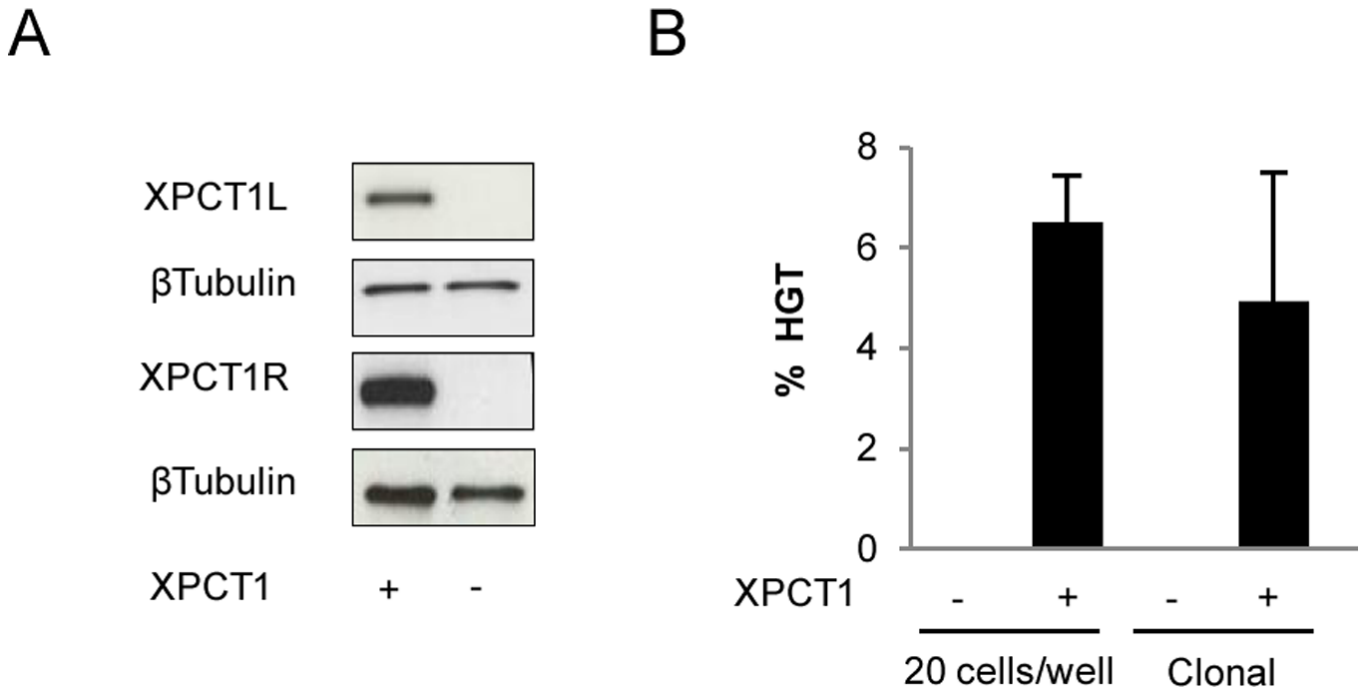
Homologous gene targeting (HGT) induced by the TALEN™ (XPCT1) in XP4PA cells. (**A**) Western blot performed on protein extracts from cells transfected with XPCT1 (+) or empty vector (−). Each monomer, XPCT1R and XPCT1L was tagged with S-tag and HA-tag, respectively. (**B**) HGT frequency was determined from XP4PA cells transfected with XPCT1 (+) or non-related TALEN™ (−) in the presence of the DNA correction matrix described in Figure 2. The transfected cells were seeded at a density of 20 cells/well or lower, enabling the formation of individual clones.

We recently reported the design of methylation-insensitive TALE nuclease using the unconventional TALE repeat N* [20]. In order to determine whether this approach could be used for the gene correction of XP4PA cells, we used the TALEN™ (XPCT1) containing the N* residue previously described to induce up to 17% of TM frequency at the methylated XPCT1t in 293-H cells. As XPCT1 targets a genomic sequence (XPCT1t) overlapping the previous meganuclease target (Figure S1A), HGT experiments were performed with the same DNA repair matrix and therefore the same screening design as for gene correction experiment using meganuclease. XP4PA cells were transfected with the DNA matrix and a plasmid encoding the XPCT1. Three days post-transfection, a fraction of cells was recovered to verify the expression of TALEN™ by western blot (Figure 3A), seeded the remaining cells at a density of 20 cells/well in a 96-well plate and characterized them three weeks later. Specific PCR screening for HGT events revealed that HGT occurred in 6.25% ±0.95 (respectively 56/192, 69/192) of transfected cells, taking into account the number of cells per well and the efficiencies of transfection and cloning (Figure 3B).

A second series of three independent experiments was then performed in which transfected cells were seeded at low density in Petri dishes allowing for clonal cell expansion. Independent clones were isolated and characterized. Under these conditions, 4.9% ±2.5 (respectively, 33/493, 49/796, 16/819) of clones were positive for HGT events (Figure 3B). As expected, no positive clone was obtained when cells were co-transfected with a non related TALEN™ and the DNA matrix. In order to determine the frequency of ΔTG correction, DNA sequencing of the genomic *XPC* gene in 45 randomly chosen HGT-positive clones was performed. The presence of the wild type sequence (i.e. the corrected sequence) was observed in 53% (24/45) of the HGT positive clones.

### Phenotypic Correction of Transfected XP4PA Cells

**Figure 4:**
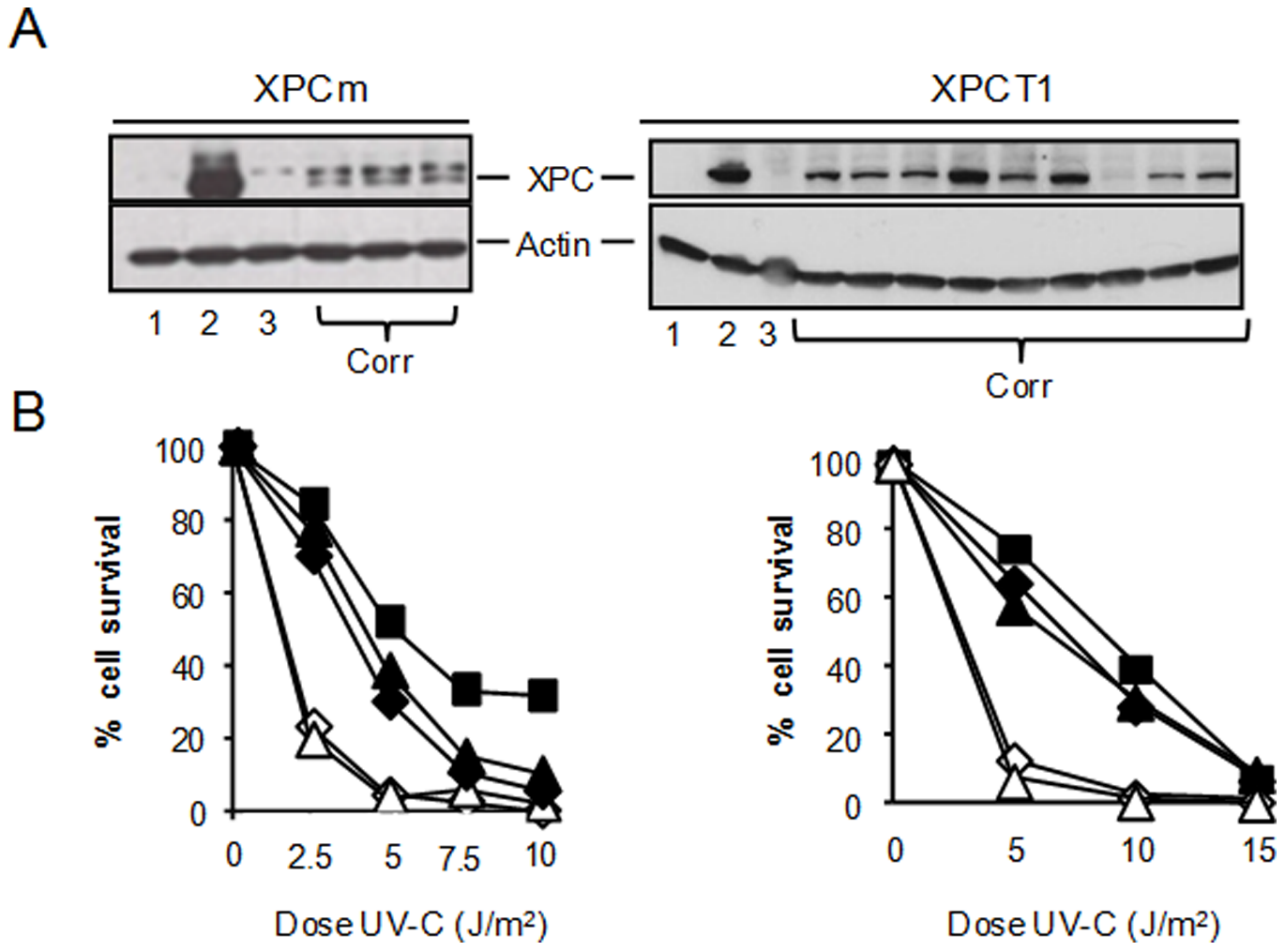
Phenotypic correction of XP4PA cells. (**A**) Western blot performed on protein extracts from clones derived from transfection with the meganuclease XPCm in the presence of demethylating treatment (left panel) or from transfection with the TALEN™ XPCT1 (right panel). XPC expression of corrected clones (Corr) was compared to negative controls, XP4PA (1), to uncorrected ΔTG clones (3), and to a positive control MRC5, proficient for XPC (2). In the left panel, an additional band is revealed by the XPC antibody. This band is most probably due to the non-specific binding of the antibody. Furthermore, this could be heightened by the 5-aza-dC treatment, as the band seems to appear only in treated samples. (**B**) UV-C survival assay on clones derived from gene correction experiment using XPCm (left panel) or using XPCT1 (right panel). The percentage of cell survival after exposure to UV-C of XPC corrected clones (closed triangle and lozenge)) was compared to two negative controls, XP4PA and uncorrected ΔTG clone (open triangle and lozenge, respectively) and one positive control MRC5 (closed square).

In order to determine whether the *XPC* gene correction induced by our engineered nucleases led to rescue of the NER pathway, we first examined the ability of the corrected clones to express the wild type XPC protein (Figure 4A and 4B). All clones showing genotypic correction displayed full-length XPC protein expression, as revealed by western blot analysis. We can observe a variable level of XPC expression between the different corrected clones, but the same variability does exist between healthy heterozygous XP-C individuals [25]. As expected, the XPC protein could not be detected in cellular clones negative for PCR-HGT or TG correction.

We also examined the stability of gene correction by keeping corrected clones resulting from transfection with the TALEN™ XPCT1. Expression level was analyzed over 3 months at population doubling (PD) 35, 65, 95 and 125 (Figure S3). The full-length XPC protein was steadily expressed over time, demonstrating the stability of the genetic correction. Finally, NER rescue was confirmed by the cell survival rate following different UV exposures (Figure 4B). One third of corrected clones expressing the XPC protein were tested for sensitivity to UV-C and showed a clear improvement in survival rate, with a behavior similar to that of the MRC5 control cell line, proficient for *XPC* (Figure 4B). This UV-C survival rescue was confirmed in corrected clones obtained from 3 independent experiments (Figure S4). As expected, clones negative for HGT events and TG correction displayed high sensitivity to UV-C. Taken together, these results provided the first demonstration of a stable correction of *XPC* mutation using sequence-specific engineered nucleases.

## Discussion

In this study, we showed that both meganuclease- and TALEN™-assisted targeted approaches allowed efficient correction of the XPC founder mutation in an XP4PA cell line derived from the fibroblasts of XP-C patients. The successful correction of *XPC*, enabled stable re-expression of the full-length XPC protein and allowed XP4PA cells to recover their fully functional NER pathway. Because of the methylation status of the sequences surrounding the XPC mutation, we opted for two independent strategies to overcome the nucleases’ sensitivity to methylation and enhance their activity *in vivo*.

In the case of the meganuclease XPCm, we used the epigenetic drug 5aza-dC to demethylate the *XPC* locus and rescue its nuclease activity. As expected, such treatments led to a significant enhancement of HGT frequency in 293-H cells (12-fold, 12% HGT, figure 1D). Although to a much lower extent, an increase of HGT frequency was also observed in XP4PA patient cell line as HGT-positive clones were obtained only after 5-aza-dC treatment. In addition, the XP4PA cells treated with 5-aza-dC displayed an altered cellular proliferation compared to their untreated counterparts. These two results might be explained by the co-existence of two deleterious factors in our experiment: the NER deficiency of XP4PA cell line and the well known ability of 5-aza-dC to alter DNA structure, especially by being inserted into DNA as a nucleotide analogue and by promoting DSB, gene expression modification, cell cycle disruption [26]. Indeed, we hypothesized that due to their NER deficiency, XP4PA cells would be unable to proficiently process altered DNA structures generated by 5-aza-dC, leading to significant cell death. Because the vast majority of corrected cells came from cells with a high level of demethylation (Figure 1B), we made the assumption that a high proportion of corrected cells were dying. Thereby, due to its deleterious effect, the use of 5-aza-dC, at least at the dose used in this study, may represent a hurdle for its application in primary keratinocytes, the relevant primary cells for genetic correction.

An alternative strategy was to use an engineered TALEN™ (XPCT1) insensitive to 5mC. This TALEN™, previously described to induce up to 17% of TM frequency at the methylated *XPC* locus in 293-H cells [20], induced about 2.5% of genetically corrected XP4PA cells in the presence of a repair matrix lacking selection marker. Because XPC is an autosomal recessive disease, a monoallelic correction of only few keratinocytes may be sufficient for clinical application providing a safe selection method is available [27].

Overall, with these two independent strategies, the TALEN™ and the meganuclease succeeded at correcting the XPC locus and restoring the full NER pathway of XP4PA cells, as shown by an UV-C survival equivalent to that of MRC5 cells. Interestingly, among the HGT-positive clones identified by HGT-specific PCR, we found that 47% still exhibited the ΔTG mutation. Such a peculiar result could be explained by the fact that the length of the conversion tract during the homologous recombination mechanism is a function of the distance from the cleavage site [28]. For a gene correction purpose, one can anticipate that the further the mutation to correct is from the cleavage site, the lower the correction frequency will be. To optimize the frequency of gene correction using nuclease-assisted targeted approaches, the engineered nuclease should be designed to cut as close as possible to the mutation to correct. Another way to improve the frequency of gene correction would be to modulate effectors such as MMR proteins, which have already been reported to decrease the gene conversion efficacy [29]. The downregulation of hMLH1 or MSH2 should increase the frequency of HGT. However, as for the use of a demethylating agent, it could be considered whether the benefits are greater than the potential adverse effects induced by MMR deficiency.

The targeted nature of nuclease-assisted gene correction offers a major advantage compared to conventional gene therapy strategies relying on retrovirus-assisted gene complementation. Because it is corrected in situ, the functional gene benefits from the natural chromosomal context and regulatory regions (endogenous promoter, terminator, and enhancer), known to play key roles in the fine tuning of gene expression. This is particularly true for the XPC promoter region, shown to contain regulatory elements located 1,700 bp upstream from the Transcription Start Site (TSS) [30]. XPC is induced following UV radiation, and the response seems to be substantial after repeated daily exposures. Likewise, our data showed that gene therapy using nucleases enabled the full-length XPC protein to be re-expressed at a level compatible with normal UV-dependent DNA damage repair. Physiological level of XPC expression is finely regulated and maintained at low background level. When XPC-GFP or HA-RAD4 were overexpressed in murine fibroblasts or in yeast respectively, a rapid degradation of these proteins by the proteasome was observed [31], [32]. Overexpression of XPC could be detrimental due to its versatile capacity to recognize physiological distortions in the DNA double helix and to bind to DNA mismatch with high affinity. Finally, we observed a steady expression of the protein at least up to 125 population doubling (Figure S4), which indicated that the expression of the corrected XPC was not down-regulated with time. This suggests that targeted nuclease approaches are unlikely to trigger epigenetic silencing of the corrected gene, as reported in complementation using retroviral approaches [33].

In summary, our work provides the first evidence that nuclease-assisted targeted approaches promote successful correction of the *XPC* founder mutation and enable restoration of the NER pathway in XP4PA cells. This study represents a strong framework for further research into xeroderma pigmentosum gene therapy.

## Materials and Methods

### Engineered Nucleases

The XPCm, RAG1m, and CAPNS1m meganucleases used in this study are derived from I-CreI and were engineered as described previously [15]. They are designed to recognize sequences within the genes XPC (NM_004628), RAG1 (NM_000448.2) and CAPNS1 (NM_001749.2), respectively. The XPCT1 TALEN™ was derived from TALE AvrBs3 and obtained from Cellectis Bioresearch [20].

### Cell Culture

Human 293-H cells (Life Technologies, Carlsbad, CA), human XP4PA and hamster CHO-KI cells (ATCC) were cultured at 37°C with 5% CO2 in complete medium DMEM for human cells and F12-K for hamster cells, supplemented with 2 mM L-glutamine, penicillin (100 IU/ml), streptomycin (100 µg/ml), amphotericin B (Fongizone: 0.25 µg/ml, Life Technologies,) and 10% FBS. XP4PA cells were derived from human dermal fibroblast from an XP-C patient carrying the homozygote mutation c.1643_1644delTG (p.Val548AlafsX25) [4], transformed by SV40 antigen T [23]. For 293-H cell transfection, 1.2 10^6^ cells were plated in 10 cm dishes. The next day, cells were transfected with 5 µg of plasmid DNA using Lipofectamine 2000 transfection reagent (Life Technologies) according to the manufacturer’s protocol. For XP4PA transfection, 1.0 10^6^ cells were transfected with 5 µg of plasmid DNA for meganuclease experiments and 10 µg or 15 µg for TALEN™ experiments, via electroporation (Lonza) using the NHDF kit and P-22 program (high viability), and seeded in 10cm dishes.

### Demethylation Treatment

Two strategies were used: for 5-aza-dC treatment, the 293-H and XP4PA cells were pre-treated 48 hours before transfection with 0.2 µM or 1 µM of 5-aza-dC (Sigma) and the treatment was maintained 48 hours post-transfection. The medium was changed every day. Two days post-transfection, genomic DNA was extracted. The monitoring of demethylation treatment was performed via bisulfite treatment, which converts cytosine (C) but not 5-methylcytosine into Uracil, using to the DNA methylation Gold Kit (Zymo Research). DNA was then amplified via PCR using specific primers (Table S1). PCR amplicons were analyzed via regular or deep sequencing using specific primers (Table S1).

### Monitoring of Nuclease Activity at Endogenous Loci

In order to evaluate the ability of nucleases to induce TM, 293-H or XP4PA cells were transfected with 3 µg of meganuclease expression vector or with 5 µg of each monomer of TALEN™ expression vector. As a control, cells were transfected with empty vector or non related TALEN™ (targeting a different genomic locus). Three days post-transfection, genomic DNA was extracted and the study targets were amplified using specific primers (Table S1) flanked by specific adapters needed for HTS sequencing, as described in Daboussi *et al* [15]. An average of 10,000 sequences per sample were analyzed**.** To evaluate the ability of nucleases to induce HGT at the *XPC* endogenous locus, cells were co-transfected with 3 µg of meganuclease expression vector and 2 µg of DNA circular matrix, or with 5 µg of each monomer of TALEN™ expression vector and 5 µg of DNA circular matrix. In 293-H cells, the matrix was composed of two homologous arms (980 bp and 1,000 bp) separated by 29 bp of an exogenous sequence. In XP4PA cells, the matrix was composed of two arms of 1.8 and 1.5 Kb homologous to the *XPC* sequences, separated by the meganuclease-recognizing site modified via silent mutation to avoid any cleavage of the matrix by XPCm (Figure 2C). The matrix was cloned in a circular plasmid. Three days post-transfection, cells were seeded at low density to form individual clones. Two weeks later, the colonies were picked up and transfered into 96-well plates for screening. DNA extraction was performed using the ZR-96 genomic DNA kit (Zymo research) according to the supplier’s protocol. The detection of targeted integration was performed via specific PCR amplification using one primer located within the heterologous insert of the DNA repair matrix and another located on the genomic sequence outside the matrix homology arms (Table S1). In pool experiments (20 cells/well or 100 cells/well), HGT frequencies were normalized to plating efficiencies (20%). Sequences of the primers used are presented in Table S1.

### Phenotypic Characterizaton of *XPC* Corrected Clones

XPC expression was revealed by western blot using an XPC specific antibody 1∶1000 (Abcam Ab6264). Actin antibody 1∶10000 (Sigma A1978) was used as a loading control**.** For the survival assay, cells were seeded at a density of 1.10^5^ cells per well in 6-well plates and exposed the following day to different doses of UV-C (254nm) at a fluency of 0.3 J/m^2^/sec. Three days post-irradiation, cells was counted and the survival frequency was determined by the ratio between irradiated and non-irradiated cells.

### Statistical Analysis

Data depicted in the Figure 1B were analysed using Chi2 test. All the other statistic analysis were performed using Student’s t-test.

## Supporting Information

Figure S1 **Engineering of nucleases with recognition of the** ***XPC*** **sequence.** (**A**) Description of the sequences targeted by the XPCm meganuclease and the XPCT1 TALEN™. The two CpG sequences are underlined. (**B**) *In vivo* cleavage activity of the XPCm, I-SceIm and RAG1 m engineered meganucleases monitored in an extrachromosomal SSA assay. TM (**C**) and HGT (**D**) frequencies were determined from 293-H cells transfected with XPCm or RAG1 m meganucleases.
(TIF)

Figure S2 **Impact of demethylating treatment on XPCt methylation status and biological consequences, in 293-H cells.** (**A**) Chromatogram showing the impact of 5-aza-dC treatment on methylating status of XPCt. Cells were grown with 0.2 µM (+) or without (−) 5-aza-dC and transfected with empty vector under the same conditions as in TM or HGT expriments. While the CpGs present in XPCt were fully methylated under non-treated conditions, the 5-aza-dC treatment induced partial demethylation as shown by the presence of a double peak. This demethylation frequency was quantified after bisulfite treament by deep sequencing (**B**). (**C**) Monitoring of non-processed DNA ends by LM-PCR in cells grown with 0.2 µM (+) or without (−) 5-aza-dC, and transfected with XPCm or RAG1 m.
(TIF)

Figure S3 **Long-term expression of the XPC protein in XP4PA corrected cells.** Two corrected clones (Corr1 and Corr2) from transfection with XPCT1 and one clone from transfection with non-related TALEN™ (control ΔTG) were kept in culture for 3 months. Protein extracts were prepared at PD35, PD65, PD95 and PD125 following transfection and XPC protein expression was monitored by western blot. XP4PA and MRC5 were used as negative and positive controls, respectively. Beta-actin was used as a loading control.
(TIF)

Figure S4 **UV-C survival assay on clones derived from gene correction experiments using XPCT1.** Clones corrected for TG mutation from experiments 1, 2 and 3 as well as uncorrected clones from experiments 1 and 2, parental cells XP4PA (negative control) or MRC5 cells, proficient for NER, were irradiated with UV-C. Three days post-irradiation, cells were counted. Cell survival was calculated as a ratio of number of cells counted after UV exposure to the number of cells counted in absence of exposure. This percentage was related to the percentage of survival of MRC5 cells.
(TIF)

Table S1 Names and sequences of oligonucleotides used to perform bisulfite sequencing analysis of XPC locus, LM-PCR and Q-PCR of XPC and RAG1 loci and to monitor TM (Targeted Mutagenesis) and HGT (Homologous Gene Targeting) events at different endogenous loci in 293-H and XP4PA cells.
(DOC)

Materials and Methods S1 **Methodologies used to perform ligation-mediated PCR (LM-PCR) and to assess meganuclease and TALENTM activities using an extrachromosomal assay.**
(DOC)
