# Supplementary figures and images for: Targeted Gene Therapy of Xeroderma Pigmentosum Cells Using Meganuclease and TALEN™
Source: PLoS One. 2013 Nov 13;8(11):e78678. doi: 10.1371/journal.pone.0078678 (PMC3827243; doi:10.1371/journal.pone.0078678)

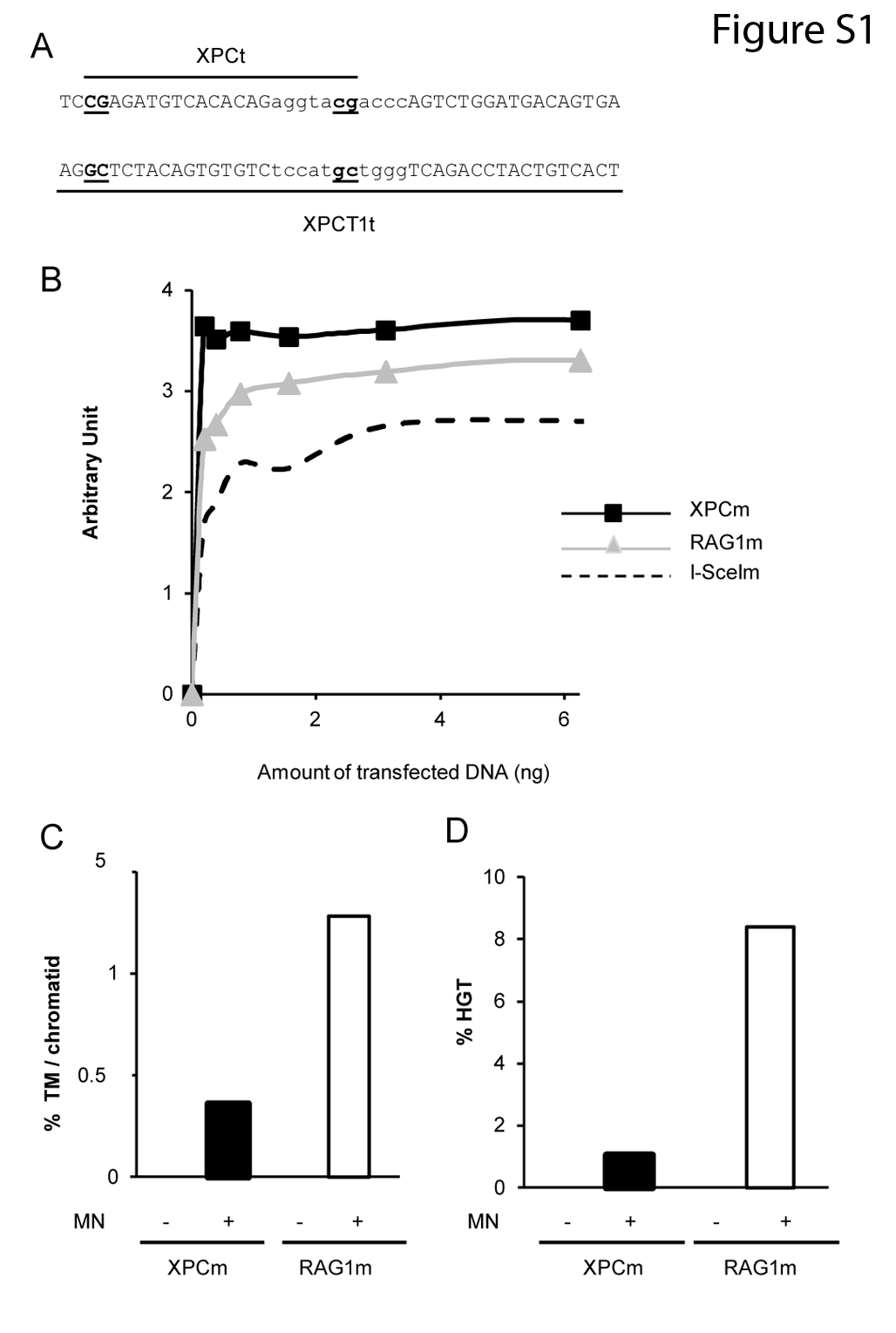

Supplement: Figure S1 — Engineering of nucleases with recognition of the XPC sequence. (A) Description of the sequences targeted by the XPCm meganuclease and the XPCT1 TALEN™. The two CpG sequences are underlined. (B) In vivo cleavage activity of the XPCm, I-SceIm and RAG1 m engineered meganucleases monitored in an extrachromosomal SSA assay. TM (C) and HGT (D) frequencies were determined from 293-H cells transfected with XPCm or RAG1 m meganucleases. (TIF) [file pone.0078678.s001.tif]

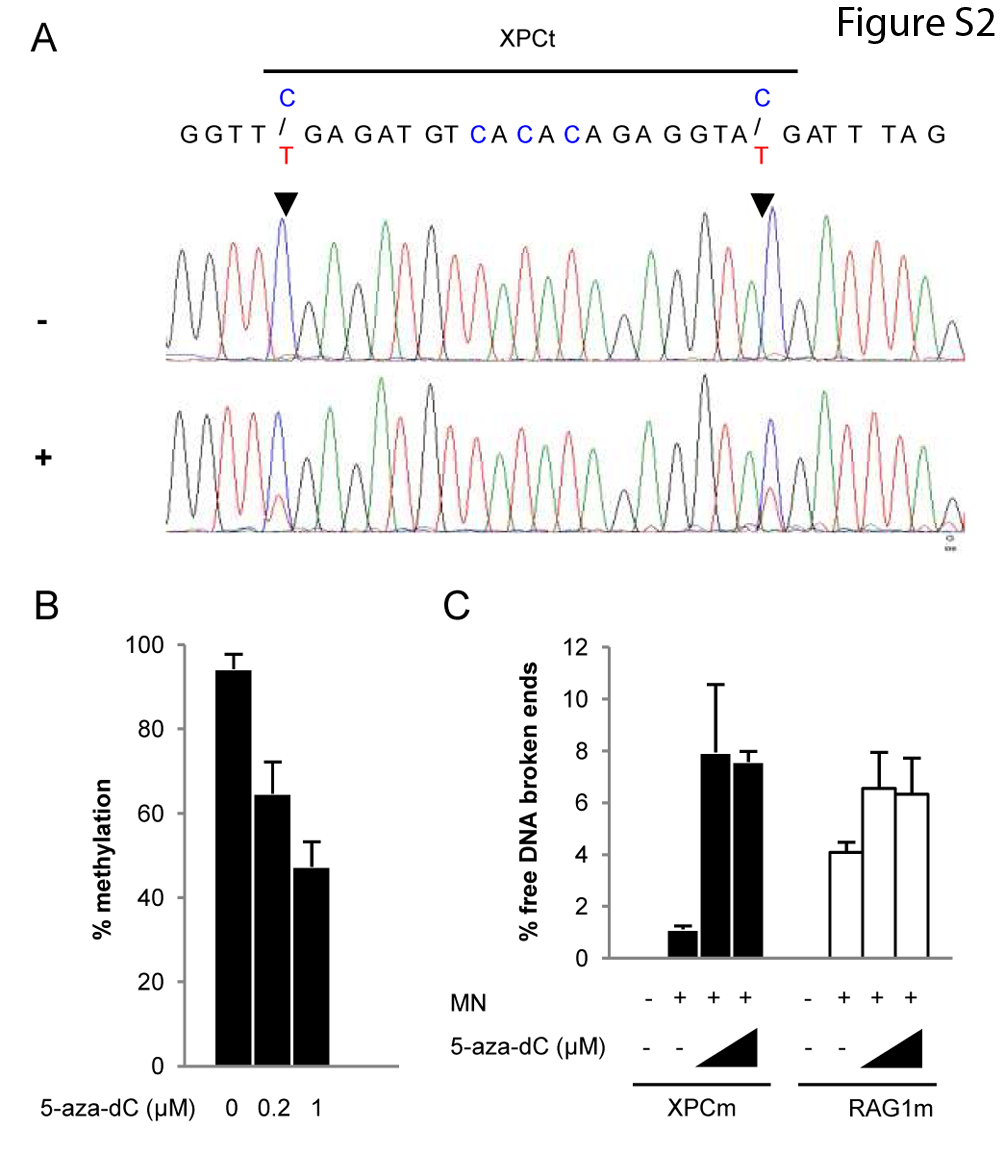

Supplement: Figure S2 — Impact of demethylating treatment on XPCt methylation status and biological consequences, in 293-H cells. (A) Chromatogram showing the impact of 5-aza-dC treatment on methylating status of XPCt. Cells were grown with 0.2 µM (+) or without (−) 5-aza-dC and transfected with empty vector under the same conditions as in TM or HGT expriments. While the CpGs present in XPCt were fully methylated under non-treated conditions, the 5-aza-dC treatment induced partial demethylation as shown by the presence of a double peak. This demethylation frequency was quantified after bisulfite treament by deep sequencing (B). (C) Monitoring of non-processed DNA ends by LM-PCR in cells grown with 0.2 µM (+) or without (−) 5-aza-dC, and transfected with XPCm or RAG1 m. (TIF) [file pone.0078678.s002.tif]

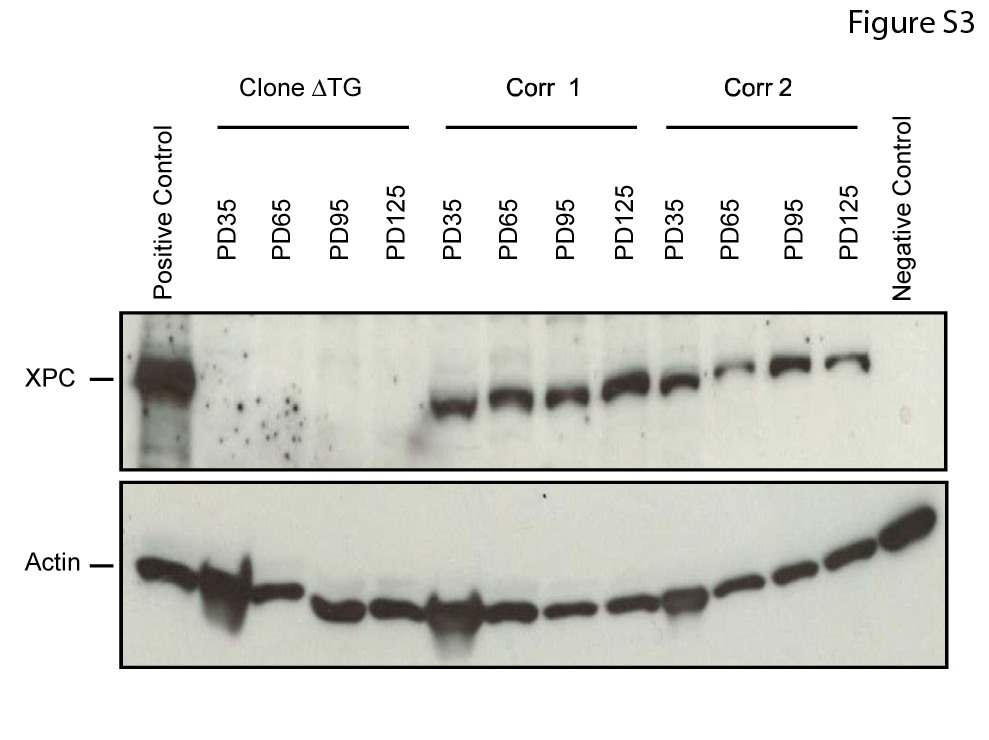

Supplement: Figure S3 — Long-term expression of the XPC protein in XP4PA corrected cells. Two corrected clones (Corr1 and Corr2) from transfection with XPCT1 and one clone from transfection with non-related TALEN™ (control ΔTG) were kept in culture for 3 months. Protein extracts were prepared at PD35, PD65, PD95 and PD125 following transfection and XPC protein expression was monitored by western blot. XP4PA and MRC5 were used as negative and positive controls, respectively. Beta-actin was used as a loading control. (TIF) [file pone.0078678.s003.tif]

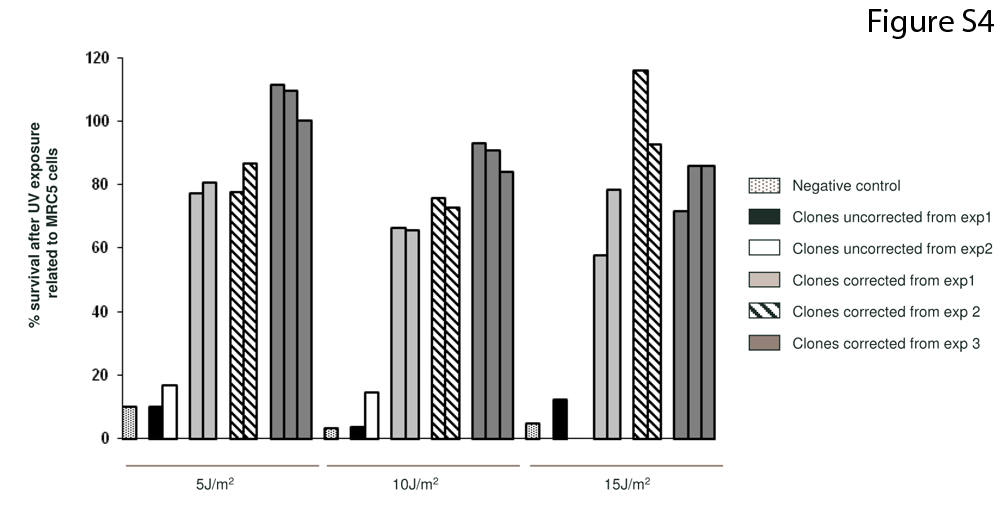

Supplement: Figure S4 — UV-C survival assay on clones derived from gene correction experiments using XPCT1. Clones corrected for TG mutation from experiments 1, 2 and 3 as well as uncorrected clones from experiments 1 and 2, parental cells XP4PA (negative control) or MRC5 cells, proficient for NER, were irradiated with UV-C. Three days post-irradiation, cells were counted. Cell survival was calculated as a ratio of number of cells counted after UV exposure to the number of cells counted in absence of exposure. This percentage was related to the percentage of survival of MRC5 cells. (TIF) [file pone.0078678.s004.tif]
