# Supplementary material for: Targeted Gene Therapy of Xeroderma Pigmentosum Cells Using Meganuclease and TALEN™
Source: PLoS One. 2013 Nov 13;8(11):e78678. doi: 10.1371/journal.pone.0078678 (PMC3827243; doi:10.1371/journal.pone.0078678)
Supplement: Table S1 — Names and sequences of oligonucleotides used to perform bisulfite sequencing analysis of XPC locus, LM-PCR and Q-PCR of XPC and RAG1 loci and to monitor TM (Targeted Mutagenesis) and HGT (Homologous Gene Targeting) events at different endogenous loci in 293-H and XP4PA cells. (DOC) [file pone.0078678.s005.doc]

| Use | Locus | Forward | Reverse |
| --- | --- | --- | --- |
| Bisulfite sequencing (amplification of locus) | XPCt | GTTGGTATAGATTAGTGGTTAGAGGTGTTTTG | CTTAAAACCCCTAACAACCAAAACCTTACC |
| Bisulfite sequencing of amplicon | XPCt | GTGGGTATGTGTAGATTGTGTGTAYGGTGTG | NA |
| LM-PCR primers for XPC and RAG1 | LM_RAG1 | TGCTGGATACGAGATGTTTACGGCTGTCTTGTGAGTGATA  CATGACGACCTATGCTCTACAAATG | |
|  | LM_XPC | TGCTGGATACGAGATGTTTACGGCTGTCTTGTGAGTGATA  GTAAACATCTCGTATCCAGCAGTGT | |
| Q_PCR primers after LM-PCR | XPCt | gcctctgatccctctgatga | gccgtaaacatctcgtatcca |
|  | TaqMan probe XPCt | atgacctatgtggtgggcat |  |
|  | RAG1t | ccataaacactgtcagaagaggaa | gccgtaaacatctcgtatcca |
|  | TaqMan probe RAG1t | catcagtgggatattgatattgg |  |
|  | Albumin | GCTGTCATCTCTTGTGGGCTGT | AAACTCATGGGAGCTGCTGGTT |
|  | TaqMan probe Albumin | CCTGTCATGCCCACACAAATCTCTCC |  |
| Monitoring of TM by deep sequencing | XPCt | AAGAGGCAAGAAAATGTGCAGC | GCTGGGCATATATAAGGTGCTCAA |
|  | RAG1t | GGCAAAGATGAATCAAAGATTCTGTCCT | GATCTCACCCGGAACAGCTTAAATTTC |
|  | CAPNS1t | CGAGTCAGGGCGGGATTAAG | CGAGACTTCACGGTTTCGCC |
| Monitoring of TM by deep sequencing after bisulfite treatment | XPCt | GTTGGTATAGATTAGTGGTTAGAGGTG | CTTAAAACCCCTAACAACCAAAACCTTACC |
| Monitoring of HGT by PCR in 293H cells | XPCt | TTAAGGCGCGCCGGACCGCGGC | GGACGCTGCACGTTCAGCTTCC |
|  | RAG1t | TCAAAAGAATTAACCCAGGCAAATTT | AATTGCGGCCGCGGTCCGGCGC |
| Monitoring of HGT by PCR in XP4PA cells | XPCt  (Screen Left) | gtcgcctactgttagacagtctgg | cgttgggttacgtccctgacc |
|  | XPCt  (Screen Right) | acgtaacccaacgatatgatcc | gatgagctcccatcagcaaccc |
| Sequencing TG correction | XPCt | ggcatcctcaagctcttcaagc | taacctgactgtgtcttggagc |

**Table S1 : Primer information**
