# Supplementary material for: Targeted Gene Therapy of Xeroderma Pigmentosum Cells Using Meganuclease and TALEN™
Source: PLoS One. 2013 Nov 13;8(11):e78678. doi: 10.1371/journal.pone.0078678 (PMC3827243; doi:10.1371/journal.pone.0078678)
Supplement: Materials and Methods S1 — Methodologies used to perform ligation-mediated PCR (LM-PCR) and to assess meganuclease and TALENTM activities using an extrachromosomal assay. (DOC) [file pone.0078678.s006.doc]

**Materials and methods supplemental**

**Extrachromosomal assay.** This assay was used to monitor the activity of the MNs in a non‑chromatinized template. CHOK1 cells were transfected with increasing amounts of nuclease‑encoding plasmids, as previously reported (21).

**Ligation-mediated PCR (LM-PCR).** 293-H cells were transfected with 3µg of RAG1m, XPCm meganucleases or empty vector. gDNA was extracted 2 days post-transfection. 1.5µg of gDNA was ligated with 1 µl of linker (1 µM) in 50 µl of buffer containing 1µl of ligase (iolabs) (400U/µl), O/N at 16°C. This linker was complementary to the 4bp 3’ overhangs cleaved by I-CreI derivatives. The reaction was stopped after inactivation of 20 mins at 65°C. Q-PCRs were performed on 50ng of ligation (QuantiFast Probe Qiagen kit). Results were normalized via Q-PCR on albumin amplicon (Table S1). Results from two experiments were expressed via fold stimulation of 5-aza-dC treatment versus untreated samples.
